# APCR, factor V gene known and novel SNPs and adverse pregnancy outcomes in an Irish cohort of pregnant women

**DOI:** 10.1186/1471-2393-10-11

**Published:** 2010-03-10

**Authors:** Sara Sedano-Balbás, Mark Lyons, Brendan Cleary, Margaret Murray, Geraldine Gaffney, Majella Maher

**Affiliations:** 1 Molecular Diagnostics Research Group, National Centre for Biomedical Engineering Science, National University of Ireland, Galway, Ireland; 2 Dept of Haematology, University College Hospital Galway, Ireland; 3 Dept of Obstetrics & Gynaecology, University College Hospital Galway, Ireland

## Abstract

**Background:**

Activated Protein C Resistance (APCR), a poor anticoagulant response of APC in haemostasis, is the commonest heritable thrombophilia. Adverse outcomes during pregnancy have been linked to APCR. This study determined the frequency of APCR, factor V gene known and novel SNPs and adverse outcomes in a group of pregnant women.

**Methods:**

Blood samples collected from 907 pregnant women were tested using the Coatest^® ^Classic and Modified functional haematological tests to establish the frequency of APCR. PCR-Restriction Enzyme Analysis (PCR-REA), PCR-DNA probe hybridisation analysis and DNA sequencing were used for molecular screening of known mutations in the factor V gene in subjects determined to have APCR based on the Coatest^® ^Classic and/or Modified functional haematological tests. Glycosylase Mediated Polymorphism Detection (GMPD), a SNP screening technique and DNA sequencing, were used to identify SNPs in the factor V gene of 5 APCR subjects.

**Results:**

Sixteen percent of the study group had an APCR phenotype. Factor V Leiden (FVL), FV Cambridge, and haplotype (H) R2 alleles were identified in this group. Thirty-three SNPs; 9 silent SNPs and 24 missense SNPs, of which 20 SNPs were novel, were identified in the 5 APCR subjects. Adverse pregnancy outcomes were found at a frequency of 35% in the group with APCR based on Classic Coatest^® ^test only and at 45% in the group with APCR based on the Modified Coatest^® ^test. Forty-eight percent of subjects with FVL had adverse outcomes while in the group of subjects with no FVL, adverse outcomes occurred at a frequency of 37%.

**Conclusions:**

Known mutations and novel SNPs in the factor V gene were identified in the study cohort determined to have APCR in pregnancy. Further studies are required to investigate the contribution of these novel SNPs to the APCR phenotype. Adverse outcomes including early pregnancy loss (EPL), preeclampsia (PET) and intrauterine growth restriction (IGUR) were not significantly more frequent in subjects with APCR compared to normal pregnant women however Pregnancy induced hypertension (PIH) was found to be associated with FVL in our study group.

## Background

APCR produces a thrombophilic state [1,2]. The combination of inherited thrombophilia and pregnancy greatly increases the risk of venous thromboembolism (VTE), which remains the commonest cause of maternal death in the developing world [3]. Thrombophilia has been linked to SNPs including Factor V G1691A (FV Leiden) (FVL), FII G20210A and MTHFR-C677T [4,5]. FVL is the commonest inherited prothrombotic SNP associated with APCR amongst Caucasians [6,7]. Other SNPs located in the factor V gene have the potential to contribute to the APCR phenotype either independently or found in association with the FVL mutation. These include Cambridge Arg ^306^, Hong Kong Arg ^306^, the Arg ^679 ^and the haplotype (H) R2 and R3 polymorphisms [8-13]. There are conflicting reports as to the association between some of these factor V gene mutations and the APCR phenotype [14,15]. A number of studies have reported the finding of APCR during pregnancy without an obvious genetic cause [16,17]. APCR in those cases, referred to as acquired APCR, is one of the mechanisms by which pregnancy induces a prothrombotic state. Acquired APCR in pregnancy is due to decreased levels of Protein S while Factor VIII levels have been reported to have an inverse correlation with APCR in pregnancy [18-21].

In this study, we screened pregnant women with APCR for known mutations and haplotypes in the factor V gene. We investigated the factor V gene in selected pregnant women with APCR for novel SNPs using GMPD, a novel mutation detection technology [22]. We determined the frequencies of adverse pregnancy outcomes in subjects with APCR based on the Classic Coatest^® ^test only and in the group with APCR based on the Modified Coatest^® ^test. The association of FVL with EPL, PET, PIH and IUGR was also examined.

## Methods

### Subjects

Written consent for samples to be collected and ethical approval for the study were obtained from the Research Ethics Committee at the University College Hospital, Galway, (UCHG) and from the 907 pregnant women included in this study attending the antenatal clinic at UCHG. Blood samples (Lithium Heparin and EDTA) were collected from these women who were ranging from 16^th ^to 24^th ^weeks gestation. The second trimester was chosen for the assessment of APC status by Coatest^® ^Classic and Modified functional haematological tests as previous studies had shown that there is little or no variation of the coagulation factors at that stage of pregnancy [23].

### Coatest^® ^haematological tests

Blood samples taken from pregnant women were drawn into evacuated anticoagulant tubes (9 volumes of blood into 1 volume of 3.2% (w/v) tri-Sodium Citrate solution). After centrifugation for 15 min at 3000 × g the plasma was stored at -80°C until they were tested using the Coatest^® ^Classic test (Chromogenix), as described by Axelsson [24]. This test measures the ratio of activated partial thromboplastin time (APTT) in the presence and absence of exogenous APC. APCR is characterised by a minimal prolongation of the APTT in response to APC and a correspondingly low ratio is determined. APCR was defined as a ratio of less than or equal to 2.1 s and APC normal as greater than 2.1 s. The 2.1 s cut off is the value established in clinical use at the hematology department in UCHG.

### Acquired APC resistance tests

Factors V, VIII and IX were determined from the classic and modified APC resistance samples and 50 APC normal samples using a one stage APTT assay on a MDA 180 coagulometer (Organon Teknika, Cambridge, UK), using factor V, VIII and IX deficient plasma's (Diagnostica Stago). Results were expressed as percentage (%) levels. Each run was controlled by the use of a normal (MDA verify1, Biomerieux) and abnormal control (coagulation control A, Technoclone GmbH).

### DNA extraction

DNA was extracted from blood samples for all APCR subjects (n = 140) using a modification of the CF(12)m-PCR protocol by Ortho-Clinical Diagnostics, Amersham, UK.

### Screening for known thrombophilic mutations

PCR-Restriction enzyme analysis (PCR-REA) was applied to identify: FVL, FV Cambridge, FV Hong Kong mutations and Haplotype (H) R2, R3 alleles in the factor V gene. DNA probe hybridisation analysis and/or DNA sequencing was applied for Arg^679 ^mutation detection. Mutations were screened in duplicate, a PCR negative control and homozygous and heterozygous controls for the different mutations were included in the screening where available. PCR amplification was performed on the GeneAmp PCR System 9600 (Perkin Elmer, USA). PCR-REA reactions were performed as referenced [8,25-28] and all PCR reagents were obtained from Promega (Madison, USA). PCR constituents were made up to a final volume of 100 μl with DNAase/RNAase free water (Sigma Aldrich, UK). Screening for the Arg ^679 ^mutation used primer set Fow 5'TgCCACAATggATTATTgTg'3 and Rev 5'AgAAACgAATTCAgTgCCAT'3 to amplify a 400 bp fragment. PCR reaction was made up to a final volume of 100 μl with DNAase/RNAase free water (Sigma Aldrich, UK). PCR included MgCl_2 _3.5 mM, 0.2 mM dNTP, in a 1 × PCR reaction buffer (10 mM Tris-HCl, 50 mM KCl, 0.1% Triton ×-100), from Promega (Madison, USA). The PCR amplification included 1 cycle of denaturation at 10 min 94°C, a second step of 35 cycles of: 1 min denaturation at 94°C, 1 min at annealing at 57°C and 1 min extension at 72°C, and a third final elongation step at 72°C for 5 min followed by holding at 4°C at the end of the reaction. A combination of 3 DNA hybridisation probes including 2 controls for the wild-type sequence (5'CATggCTACACggAAAATgCATg'3, 5'gTTAACTTCCATgAATTCTAgTCCAAg'3) and a mutant probe (5'gTTAACTTCgATgAATTCTAgTCCAAg'3) were designed from factor V sequence (GenBank Accession number AY364535) to identify mutations in the Arg ^679 ^cleavage site of the factor V gene following amplification with the Arg ^679 ^site primers.

### Adverse pregnancy outcomes

An evaluation of the outcomes of pregnancy including; EPL, PET, PIH and IUGR was performed by examination of maternity records at UCHG for the study subjects The criteria for diagnosis were: EPL-any pregnancy loss before 12 weeks gestation (spontaneous abortion or ectopic). PET-BP (Blood pressure) ≥ 140/90 mmHg, +1 proteinuria and oedema. PIH-BP ≥ 140/90 with no proteinuria in accordance with the Official Journal of the International Society for the Study of Hypertension in Pregnancy. IUGR-is defined as fetal growth of less than 5^th ^percentile for gestational age [29].

### Statistical analysis

The Chi-Square test from SPSS version 17.0 software was performed to test if there was a significant difference in the proportion of the different outcomes in the subjects with APCR with only the Classic Coatest^® ^test (n = 105) compared to the subjects with APCR based on the Modified Coatest^® ^test (n = 35). The same statistical analysis was also performed to compare each adverse outcome type in FVL subjects (n = 29) with subjects without FVL (n = 878). Ninety-five percent Confidence intervals (CI) were determined. The Kruskal-Wallis statistical test http://www.graphpad.com was applied to compare levels of FV, FVIII and FIX in APCR and APC normal pregnant subjects. P values < 0.05 were considered significant.

### Screening for novel thrombophilic mutations by GMPD

Five patients with APCR and 1 APC normal control subject were selected for scanning for SNPs in the factor V gene by glycosylase mediated polymorphism detection (GMPD) analysis. The normal dTTP nucleotide is partially modified by introducing dUTP during PCR amplification for the GMPD. PCR primers (Additional file 1, Table S1) to amplify all 25 exons of factor V gene were designed using the Primer3 program http://www-genome.wi.mit.edu/cgi-bin/primer/primer3_www.cgi and the factor V sequence GenBank Accession number Z99572. PCR was optimised to determine optimal MgCl_2 _and annealing temperature conditions for amplification of factor V exons (Additional file 1, Table S1). Primers were synthesised by TIB MOLBIOL, (Germany) with a 5' fluorescent label modification, 6-FAM (6-carboxyfluorescein) on the forward primer and a 5'HEX (hexachlorinated analogue of 6-FAM) modification on the reverse primer to perform GMPD analysis on both strands of the PCR products. PCR reactions were performed in duplicate in 20 μl volume and included approximately 30-50 ng genomic DNA, 1.5 U *Taq *polymerase, 12.5 pmol of each appropriate labeled PCR primer, MgCl_2 _concentration as appropriate to the PCR primer set pair (Additional file 1, Table S1) and 0.2 mM of each of dATP, dCTP, dGTP, and 1:60 ratio of dTTP: dUTP in distilled H_2_O. PCR product (10 μl) was mixed with 1 μl of 0.5 U/μl exonuclease I (New England Biolabs, UK) and incubated at 80°C for 15 min. The exonuclease I treated PCR product mix was purified on an A S300HR clean-up column (Amersham Pharmacia Biotech, England) following manufacturers instructions. The purified PCR products were incubated at 37°C for 30 min with one microlitre of 0.5 U/μl uracil DNA-glycosylase (New England Biolabs, UK) to cleave the N-glycosidic bond between the base uracil and the sugar deoxyribose in the DNA molecule. This reaction generates an apyrimidinic (AP) site. The AP site was cleaved chemically by adding 1 μl of 0.6 M NaOH (Sigma Chemical, USA) (final concentration of 0.05 M NaOH) to the reaction mix and incubated at 95°C for 15 min. To neutralise the reaction, 1 μl of 0.45 M Tris base (Sigma Chemical, USA) (final concentration of 0.03 M) was added. One microlitre of GeneScan internal standard (ROX-500; PE, Applied Biosystems, USA) was also added to each sample. Prior to analysis by electrophoresis on a 6% PAGE (7 M urea), denaturation of samples was performed by adding 1 μl of deionised formamide (Applied Biosystems, USA) and 2 μl of 50 mg ml^-1 ^blue dextran dye in 25 mM EDTA (Sigma Chemical, USA). The mixture was heated at 95°C for 5 min prior to the run. The ABI PRISM 377 automated DNA sequencer (Applied Biosystems, PE, USA) was set at 3 kV/h for 6 h using filter A at 2400 scans/h.

### DNA sequencing

PCR products from selected subjects screened for the Arg ^679 ^mutation and PCR products that were identified to contain an SNP by GMPD were sequenced. PCR products were purified using the QIAprep Spin Miniprep Kit (Qiagen Ltd, UK) according to the manufacturers instructions and sent to a commercial sequence service provider (MWG Biotech, Germany) for sequencing.

### Analysis of amino acid changes resulting from detected SNPs

The mRNA sequence of factor V (6913 bp nucleotides, GenBank Accession number NM_000130) was translated into its protein product using the internet translation tool at http://us.expasy.org/tools/dna.html which identified each amino acid and its corresponding codon sequence. The nucleotide and amino acid sequences for each exon were compared with the sequences reported by Jenny *et al*. (1987). Other sequences were also included to compare the nucleotides and amino acids changes at positions where SNPs were identified in this study, these sequences included the following GenBank Accession numbers: NP_000121, NM_000130, M16967, Z99572 and XM_001575.

## Results

### Description of study cohort

We determined that 16% (n = 140) of pregnant women tested had APCR, 12% of whom had APCR based on Classic Coatest^® ^test and 4% had APCR with the Classic and Modified Coatest^® ^tests. One subject had APCR with the Modified Coatest^® ^test only. Eighty-nine Classic APCR subjects had no known mutations in the factor V gene. Sixteen subjects had the FV HR2 haplotype. Thirty-four subjects were identified to have APCR based on the Classic and Modified test, 29 of whom had the FVL mutation. One subject with FVL also had the FV Cambridge-G1091C mutation (Table 1).

Measurement of Factor V, Factor VIII and Factor IX in subjects with classic APCR and modified APCR determined the levels of Factor V to be significantly decreased and the levels of Factors VIII and IX to be significantly increased compared to normal pregnant controls.

**Table 1:** Distribution of known mutations in the Factor V gene

**APCR with Classic Coatest**^® ^**test only n = 105**		
**Frequency %**	**n**	**mutation**
15	16	ht (H) R2
85	89	no mutations
**APCR with Classic and Modified Coatest**^® ^**test n = 34**		
**Frequency %**	**n**	**mutation**
85	29	hz FV Leiden
3	1	hz Cambridge*^1^
3	1	hz (H) R2
14	4	no mutations
**APCR with Modified Coatest**^® ^**test only n = 1**		
**Frequency %**	**n**	**mutation**
100	1*^2^	no mutations

Abbreviations: (n) number of subjects, (ht) heterozygotes, (hom) homozygotes, (H) haplotype.

*^1 ^n = 1 had hz Cambridge and FVL mutations simultaneously.

* ^2 ^Subject was not included in the scanning for novel SNPs as insufficient DNA sample was available for the study.

### Novel SNPs identified in APCR subjects with Modified Coatest^® ^test and without FVL

We selected five subjects for investigation for novel SNPs in the factor V gene because they had APCR with the Modified Coatest^® ^test and no FVL or other known mutations in the APC cleavage sites of factor V gene. Subject 1, a carrier of (H) R2 allele had 9 SNPs in the factor V gene at nucleotide positions 1216 in exon 8, 2325, 2297/8, 3015, 4038, 4047 and 4648 in exon 13 and 5380 in exon 16; subject 2 had 10 SNPs in the factor V gene at nucleotide positions 1216/7, 1219 and 1226 in exon 8, 1977 in exon 12 and 2904, 3894, 4040/1, 4185 in exon 13; subject 3 had 5 SNPs in the factor V gene at nucleotide positions, 2663, 2684, 2710, 2887 and 4125 in exon 13; subject 4 had 5 SNPs in the factor V gene at nucleotide positions 2127, 2298, 2391 and 2893/4 in exon 13 and subject 5 had 4 SNPs in the factor V gene at nucleotide positions 2863, 2883, 2884/5, in exon 13.

In total, 33 polymorphisms were identified, in 5 cases (2297/8, 1216/7, 2893/4, 2884/5, 4040/1) two SNPs were identified in the same codon. All the SNPs were confirmed in duplicate samples and by comparison to GenBank sequence NM_000130 and other available factor V sequences. Table 2 summarises the SNPs detected by GMPD and identified by DNA sequencing in this study.

**Table 2:** SNPs identified in the FV gene by GMPD

**Non-silent SNPs**					
**SNP identified by GMPD**			**NM_000130 sequence**		
**codon**	**amino acid**	**Exon**	**nucleotide**	**codon**	**amino acid**
TTC	Phe [F]	13	2127	TTg	Leu [L]-651
AAC	Asn [N]	13	2297/8	ATT	Ile [I] -708
AAA	Lys [K]* ^1^	13	2663	AgA	Arg [R]-830
CAT	His [H]* ^2^	13	2684	CgT	Arg [R]-837
AAg	Lys [K]* ^3^	13	2863	gAg	Glu [E]-897
TCT/TCA	Ser [S]	13	"2884/5"	TTA	Leu [L]-903
TTT/	Phe [F]/	13	"2884/5"	TTA	Leu [L]-903
gTC	Val [V]	13	2887	CTC	Leu [L]-904
ATA/	Ile [I]/	13	"2893/4"	AAA	Lys [K]-906
CAA/	Glu [E]/	13	"2893/4"	AAA	Lys [K]-906
CTA	Leu [L]	13	"2893/4"	AAA	Lys [K]-906
AAg	Lys [K]	13	2904	AAC	Asn [N]-909
AAT	Asn [N]	13	4040/1	ggT	Gly [G]-1289
ATA	Ile [I]	13	4047	ATg	Met [M]-1291
Cgg	Arg [R]	13	4125	CAg	Glu [Q]-1308
TCA	Ser [S]	13	4648	ACA	Thr [T]-1492
Atg	Met [M]* ^4^	16	5380	gTg	Val [V]-1736
Ggg	Gly [G]	8	1216	Agg	Arg [R]-348
CAg	Gln [Q]	8	1216/7	Agg	Arg [R]-348
CCT	Pro [P]	8	1219	TCT	Ser [S]-349
CTT	Leu [L]	8	1226	CAT	His [H]-350
CAA	Gln [Q]	12	1977	CAC	His [H]-601
**Silent SNPs**					
**SNP identified by GMPD**			**NM_000130 sequence**		
**Codon**	**amino acid**	**exon**	**nucleotide**	**codon**	**amino acid**
ATA	Ile [I]	13	2298	ATT	Ile [I]-708
AAC	Asn [N]	13	2325	AAT	Asn [N]-712
TCA	Ser [S]/	13	2391	TCg	Ser [S]-739
gAA	Glu [E]	13	2710	CAA	Gln [Q]-846
CTA	Leu [L]	13	2883	CTg	Leu [L]-902
CCT	Pro [P]	13	3015	CCC	Pro [P]-947
TCC	Ser [S]	13	3894	TCT	Ser [S]-1240
CTT	Leu [L]	13	4038	CTC	Leu [L]-1288
ACT	Thr [T]	13	4185	ACC	Thr [T]-1337

Notes:

Nucleotide and amino acid positions presented in this table correspond to GenBank sequence NM_000130. When (/) appears between nucleotide bases, it indicates that the SNPs were found in the same codon. The (" ") indicate that there were identified possible amino acids encoded by the SNPs at that nucleotide position. The amino acids at codon residues 348 and 708 appear twice in the table as they were found in different subjects with different SNPs. Abbreviations: (A) adenine, (G) guanine, (T) thymine and (C) cytosine.

*^1 ^[R] in NP_000121, NM_000130, M16967 and [K] in Z99572, XM_001575.

*^2 ^[R] in NP_000121, NM_000130, M16967 and [H] in Z99572, XM_001575.

*^3 ^[E] in NP_000121, NM_000130, M16967 and [K] in Z99572, XM_001575.

*^4 ^[V] in NP_000121, NM_000130, M16967 and [M] in Z99572, XM_001575.

### Adverse outcomes

Table 3 shows the type and number of adverse pregnancy outcomes in subjects with APCR, based on the Classic Coatest^® ^test (n = 105) only, compared to the subjects with APCR based on the Modified Coatest^® ^test (n = 35). Table 3 also shows a comparison of adverse outcomes in FVL subjects (n = 29) with subjects without FVL (n = 878). No significant difference was found in the frequency for EPL, PET or IUGR between the groups compared. However PIH had a significantly higher frequency in subjects with APCR based on the Modified Coatest^® ^test (Chi-Square p value 0.018) compared to subjects with APCR based on the Classic Coatest^® ^test only. PIH frequency was also significantly different (Chi-Square-p value 0.004) in FVL subjects compared to the subjects with no FVL.

**Table 3:** Adverse obstetric outcomes

**Subjects identified (n)**	**Type and number of adverse pregnancy outcomes**			
**APC resistance n = 140**	**EPL**	**PET**	**PIH**	**IUGR**
**Only Classic n = 105**	29/105	2/105	5/105	1/105
**Modified n = 35**	9/35	1/35	6/35	0/35
**Chi-Square p value**	.826	.736	.018	.562
**Subjects identified (n)**	**Type and number of adverse pregnancy outcomes**			
**Total cohort n = 907**	**EPL**	**PET**	**PIH**	**IUGR**
**No FVL n = 878**	226/878	41/878	43/878	21/878
**FVL n = 29**	8/29	1/29	5/29	0/29
**Chi-Square p value**	.832	.748	.004	.396

## Discussion

Factors that are thought to contribute to APCR in the general population and during pregnancy include defects in the Factor V protein, most frequently caused by the Leiden mutation [1,30]. Several reports have shown however that between 5-10% of APCR does not involve the FVL and the cause of APCR in these cases is not known [1,8,31-33]. APCR in these cases may be the result of acquired factors including pregnancy. The frequency of acquired APCR in our study group, indicated by women showing APCR with Classic Coatest^® ^test tested in the second trimester only was 12%. This correlates with various studies showing reduced APC ratios in the Classic Coatest^® ^test during pregnancy [34,35]. Testing of true APCR status in the study cohort by a second measurement of APC at pre-pregnancy or 8 to 12 weeks post-pregnancy or more frequently during pregnancy was not performed and this is a major limitation of the current study. Mahieu *et al*. (2007) recently reported an inverse correlation between increasing Factor VIII and decreasing APC sensitivity ratio in second trimester pregnant women. Increasing Factor VIII has been linked to early pregnancy loss [36]. In our study we found the levels of Factors VIII to be significantly increased in APCR subjects compared to normal pregnant controls. Overall FVL frequency in our study group (n = 907) was 3.1% which is slightly lower than the range of 4-7% reported in previous Irish studies [37,38] but within the range of prevalence of FVL in the general Caucasian population [39]. Six subjects who had APCR with the Modified Coatest^® ^test did not have the FVL suggesting that Coatest^® ^test can give false positive results.

APCR has been considered a risk factor for thrombosis in pregnancy and it has been suggested that pregnant women who have had previous thrombotic events or who have a family history of the FVL mutation should be screened for the FVL mutation [40]. Other mutations in the factor V gene, particularly at the other cleavage sites, Arg ^306 ^and Arg^679 ^of Factor V have the potential to contribute to the APCR phenotype [8,41]. No mutations have been identified to date at Arg^679 ^in this or other studies [42], Van der Neut Kolfschoten, 2005 (personal communication).

One subject in our APCR cohort had 2 mutations in the factor V gene; FVL and FV Cambridge G1091C, this subject developed PIH during pregnancy. A recent Spanish study identified the FVL and FV Cambridge mutations in a subject with a family history of thrombosis [43].

Haplotypes (H) R2/R3 have also been associated with mild APCR when found in combination with the FVL [12,15,42]. In our study, the (H) R2 allele was found in 12% of the APCR cohort. We did not identify the (H) R3 allele mutation in the APCR group which is consistent with published literature [8]. There have been a number of other mutations identified in other studies on the factor V gene however they do not appear to contribute significantly to APCR in Caucasian populations, for example, R485K and I359T [9,44,45].

It was previously speculated that APCR may be caused by a novel defect in the factor V molecule [46]. We identified 33 SNPs including 20 novel SNPs in 5 APCR subjects scanned for SNPs by GMPD. A number of the SNPs (nucleotide positions 2663, 2684, 2863, 5380) were identified previously in GenBank sequences of Factor V (NP_000121, NM_000130, M16967, Z99572 or XM_001575). The identification of these SNPs by GMPD validated the SNP scanning technique we used.

Previous studies defined the R2-H1229A haplotype polymorphism as a combination of mutations including, C2298T, T2325C, A2379G, A2391G and A4070G (HR3) in exon 13 and A5380G in exon 16 [11,12,47]. In our study, the heterozygous carrier of R2 allele-H1229A was determined to have T2298A, T2325C, and G5380A SNPs. The haplotype (H) R2 has also been linked to altered levels and ratios of Factor V1 and Factor V2 [11,45]. We also found G2391A SNP, previously associated with the HR2 haplotype, in one subject. Four of the novel SNPs identified in this study are located in exon 8 while most exons have no SNPs identified in this study. This may suggest that Factor V amino acids 348-350 have an association with APCR.

In a study performed by Kostka *et al*. (2000), the presence of missense mutations in the first 1200 bp of the Exon 13 (B-domain) of the factor V gene was investigated in a group of APCR patients who did not carry the FVL mutation. Four silent mutations, C2298T, T2325C, A2379G, A2391G and four missense mutations; A2540C, A2663G, A2684G, A2863G were identified in patients with thrombosis and normal controls [48]. In our study the SNP T2325C was identified and SNPs at nucleotide positions 2298(T2298A), 2391(G2391A), 2663(G2663A), 2684(G2684A) and 2863(G2863A) were also identified. SNP T3894C identified in our study was also previously identified in a study performed on factor V levels in plasma by Lunghi *et al*. (1996). Twenty-six of the SNPs identified in this study, were in exon 13 which encodes most of the B domain of the FV protein. This high frequency of SNPs in exon 13 has been previously reported [26,49]. The large heavily glycosylated B-domain, spanning amino acids 710-1545, was previously reported not to be essential for the cofactor activity of the FV glycoprotein as it is released during the activation of FV [50,51]. However, other studies have postulated that the B domain may be involved in other coagulation related functions including the proteolytic activation of thrombin. A previous study showed decreased activation of thrombin when the B domain of FV was completely deleted [52]. Kostka *et al*. (2000) suggested that the B domain of FV is of vital importance in APC cofactor activity in the clotting cascade, and that mutations in this domain may contribute to an ineffective APC response. The C-terminal part of the B-domain (residues 1477-1545) was suggested to be crucial for the APC-cofactor function [53]. It has also been suggested that a region of FV B domain may function in Factor VIII (a) inactivation by APC through the FV-PS-APC system [54-56]. Mutations in exon 13 may disrupt the calcium-binding site in the FV protein leading to a loss of the high-affinity (calcium-dependent) interaction of the heavy chain and light chain of FVa potentially influencing the dissociation of the heavy chain and light chain [57-59].

Dawood *et al*. (2007) found significant increased frequency of SNPs in the factor V gene in the women with acquired APCR and recurrent miscarriage in comparison with their control groups [60]. In our study we identified several SNPs in the Modified APCR subjects with no FVL. The finding of 20 novel factor V gene SNPs in these subjects is interesting. These SNPs may have an effect on FV levels rather than directly causing APCR. A future study screening the normal population would be useful to determine if these SNPs are common in the population. It may be that these SNPs are common in Irish people but rarely found in other populations.

The total frequency of adverse outcomes was not significantly different in the group with APCR based on the Modified test compared to the pregnant group with classic APCR, which is consistent with the findings of recent studies [61,62]. Comparison of the frequency of each adverse outcome individually determined a statistically significant higher prevalence of PIH in subjects with APCR with the Modified Coatest^® ^test and in subjects with FVL compared to subjects with no FVL. Of 48 subjects from the total study cohort with PIH, 3 subjects had PET, two of whom had APCR and only one had FVL. PET in this cohort is associated with changes to coagulation system other than those caused by FVL. FVL was significantly associated with PIH in a German study investigating 70 women with severe PIH, 15 of whom had PET [63]. In contrast, a study of 100 pregnant women with PIH in Turkey did not find a significant association between FVL and PIH [64]. Discrepancies as to the association of adverse outcomes with FVL can be a consequence of study size, presence of additional thrombophilic factors, different study design and methodology or differences in the genetic population studied [65,66].

## Conclusions

PIH appeared to be associated with FVL whereas no association was found between FVL and EPL, PET or IUGR in our pregnant study group. We identified 6 pregnant women with APCR without FVL in our modified APCR group. Further studies are required to investigate the contribution of novel SNPs identified in this study to the APCR phenotype.

## Competing interests

The authors declare that they have no competing interests.

## Authors' contributions

SSB: Contributed to the overall study design, in particular the design of experiments required to undertake the study as well as their performance. SSB took the lead in writing this manuscript. ML: Determined the APC ratio from the pregnant women included in the study using the functional haematological tests; Coatest^® ^Classic and Modified tests at University College Hospital Galway (UCHG). He also measured levels of factors V, VIII and IX in the APCR group and provided discussion and support to the manuscript preparation. BC: Provided oversight to the tests performed at UCHG for the study and discussion and interpretation of the results of the study. MM: Contributed to the overall design of the study and made her lab facilities and personnel at UCHG available to participate in the study. GG: Organised the ethical approval for the study and the sample collection for the study. She contributed to the study design and analysis of the scientific results of the study. MMah: Contributed to the overall study design, the organisation and execution of the planned experiments. She supervised and contributed to the preparation of this manuscript. All authors read and approved the final manuscript.

## Pre-publication history

The pre-publication history for this paper can be accessed here:

http://www.biomedcentral.com/1471-2393/10/11/prepub

## Supplementary Material

Additional file 1 **Primers used for GMPD**. PCR primers for the GMPD to amplify all 25 exons of factor V gene. These were designed using the Primer3 program http://www-genome.wi.mit.edu/cgi-bin/primer/primer3_www.cgi and the factor V sequence GenBank Accession number Z99572A. Click here for file
