# Supplementary material for: APCR, factor V gene known and novel SNPs and adverse pregnancy outcomes in an Irish cohort of pregnant women
Source: BMC Pregnancy Childbirth. 2010 Mar 10;10:11. doi: 10.1186/1471-2393-10-11 (PMC2853494; doi:10.1186/1471-2393-10-11)
Supplement: Additional file 1 — Primers used for GMPD. PCR primers for the GMPD to amplify all 25 exons of factor V gene. These were designed using the Primer3 program http://www-genome.wi.mit.edu/cgi-bin/primer/primer3_www.cgi and the factor V sequence GenBank Accession number Z99572A. [file 1471-2393-10-11-S1.DOCX]

**Additional file 1, Table S1: Primers used for GMPD**

| **Primers used for GMPD from exons 1 to 12 and 14 to 25 of factor V gene** | | | | |
| --- | --- | --- | --- | --- |
| **Exon**  **name** | **Olignucleotide-Primer sequence 5’**  **Forw 5’ 6-FAM /Rev 5’ HEX** | **Annealing**  **Tª (ºC)** | **MgCl_2_**  **(mM)** | **Size**  **(bp)** |
| Exon1 F | 5’CTTCACCTgCAgTAAAACAgT3’ | 55 | 4.5 | 522 |
| Exon1R | 5’CAAAgggAATgTTCTCTAAAAg3’ |  |  |  |
| Exon 2 F | 5’TAgTTATCACAACAggATATTTTTg3’ | 55 | 4.5 | 506 |
| Exon 2R | 5’gAAATTTTCAATAATATgAACAAgg3’ |  |  |  |
| Exon 3 F | 5’CTTCTAgATTgAAAATAAgTgAg3’ | 50 | 4.5 | 496 |
| Exon 3 R | 5’CCAAACATTAAAgAgTAAgAAC3’ |  |  |  |
| Exon 4 F | 5’TgTTATTCTTAAgATCACCATgT3’ | 50 | 4.5 | 529 |
| Exon 4 R | 5’gATgTACAAgAAgTTACCAAgAT3’ |  |  |  |
| Exon 5 F | 5’CCTAgAACCATTATTACTTgAT3’ | 47 | 4.8 | 502 |
| Exon 5 R | 5’ACATTTCTTCTCTAgCTTCTAA3’ |  |  |  |
| Exon 6 F | 5’CATCTCTAAgCTCTgTTgTTA3’ | 55 | 5.3 | 611 |
| Exon 6 R | 5’ATATCTAAgTTTggTTgTTAgg3’ |  |  |  |
| Exon 7 F | 5’ACCCACAgAAAATgATGCCCAg3’ | 50 | 3.5 | 228 |
| Exon 7 R | 5’TCTTgAACCTTTgCCCA3’ |  |  |  |
| Exon 8 F | 5’ATgTTTAAgCACAAgggTTTCC3’ | 55 | 5.3 | 508 |
| Exon 8 R | 5’TCCCCgTATTCTATCCCAATTA3’ |  |  |  |
| Exon 9 F | 5’gATgAggggAggTTACTgC3’ | 51 | 4.8 | 484 |
| Exon 9 R | 5’CTAgAggCCACTATgACACT3’ |  |  |  |
| Exon 10 F | 5’ggTCCAgCgAAAGCTTATTTATT3’ | 60 | 1.2 | 387 |
| Exon 10 R | 5’TgCCCCATTATTTTAgCCAggAg3’ |  |  |  |
| Exon 11 F | 5’TTTCCCTATTgCTTgCTTTTgT3’ | 57 | 3.8 | 470 |
| Exon 11 R | 5’gACCTCTTgCTTAAAAATgTTgC3’ |  |  |  |
| Exon 12 F | 5’TAACATggAgACATTTATCTTAg3’ | 55 | 3.8 | 541 |
| Exon 12 R | 5’gTATATTTTTACTTCCTACTTTgC3’ |  |  |  |
| Exon 14 F | 5’TCTCTATgCTAgATgCTATATTT3’ | 50 | 3.8 | 502 |
| Exon 14 R | 5’TTTCCACAgATTATATAgTTTTC3’ |  |  |  |
| Exon 15 F | 5’CTTATATCTTAgCTCCgATTTTAT3’ | 50 | 3.8 | 506 |
| Exon 15 R | 5’gTTATCATAAATgCAgACTgTTA3’ |  |  |  |
| Exon 16 F | 5’gAATACAACAgAATTAggTgATAA3’ | 50 | 3.8 | 572 |
| Exon 16 R | 5’TCTAATCTTgTgAATATCTAAgg3’ |  |  |  |
| Exon 17 F | 5’CTCTCACTAATgTgACATACAA3’ | 49 | 3.8 | 522 |
| Exon 17 R | 5’CATTTTTgTACTCCgTATATACTTT3’ |  |  |  |
| Exon 18 F | 5’AATggTTAAATTCTATTTgAAAgC3’ | 51 | 3.8 | 511 |
| Exon 18 R | 5’gAAAATTTCTTATATgCACTTgTC3’ |  |  |  |
| Exon 19 F | 5’CTTgTTAgTgCATTTACCATTTTAT3’ | 56 | 3.8 | 439 |
| Exon 19 R | 5’CTTTAgCTCCTACCATAgTgTT3’ |  |  |  |
| Exon 20 F | 5’AgATTTCATCATCAAggACTg3’ | 46 | 3.8 | 458 |
| Exon 20 R | 5’CAAgATATTATTAgAACCAACC3’ |  |  |  |
| Exon 21 F | 5’TTgAgTTgggATTCAAATTTAggTA3’ | 56 | 3.8 | 490 |
| Exon 21 R | 5’CTTTTCTCCATATgACCCTTAgAAA3’ |  |  |  |
| Exon 22 F | 5’ACCAAATTgATTTAgTTTggACC3’ | 56 | 3.8 | 504 |
| Exon 22 R | 5’CTATATAATCCAggCACTTTCTT3’ |  |  |  |
| Exon 23 F | 5’gTAATATTATTTCTTATCTCCTCTg3’ | 48 | 7 | 478 |
| Exon 23 R | 5’ggTTTAAATCAAgACTAgACC3’ |  |  |  |
| Exon 24 F | 5’TAggAgACTgTgAATCCTAAg3’ | 51 | 3 | 505 |
| Exon 24 R | 5’gAgAAATAAAACAAACCAAAACAgA3’ |  |  |  |
| Exon 25 1F | 5’gATTACTTgAgAggAgTTTAAg3’ | 45 | 3.8 | 543 |
| Exon 25 1R | 5’gTTCTTgATATTTTCCTACCTgTAT3’ |  |  |  |
| Exon 25 2F | 5’TCAAAAACCCCTggAAgAgA3’ | 50 | 2.7 | 534 |
| Exon 25 2R | 5’TCAATggggCTACTggAAAg 3’ |  |  |  |
| **Primers used for GMPD on exon 13 of factor V gene** | | | | |
| **Exon**  **name** | **Olignucleotide-Primer sequence 5’**  **Forw 5’ 6-FAM /Rev 5’ HEX** | **Annealing**  **Tª (ºC)** | **MgCl_2_**  **(mM)** | **Size**  **(bp)** |
| Exon 13 1F | 5’AgCAAAAAgCTgAggCTgAA3’ | 57 | 3 | 385 |
| Exon 13 1R | 5’gAgAAggggCTTTCTgAggT3’ |  |  |  |
| Exon13 2F | 5’TCTTCCACAgCAgAgCATTC3’ | 57 | 4.5 | 303 |
| Exon 13 2R | 5’CCTCATTCTggAAggAgAACC3’ |  |  |  |
| Exon 13 3F | 5’AACAAgCCTggAAAgCAgAg3’ | 57 | 4.5 | 323 |
| Exon 13 3R | 5’AgTgAggCTATCCAgCCAAA3’ |  |  |  |
| Exon 13 4F | 5’gACCCCAgTCACAgATCCTC3’ | 57 | 1.5 | 285 |
| Exon 13 4R | 5’ATggCTgAggTCTggAgAAA3’ |  |  |  |
| Exon 135F | 5’CCAgCCATACAACCCTTTCT3’ | 57 | 1.5 | 301 |
| Exon 13 5R | 5’TCAAggTCTggAggAggTgA3’ |  |  |  |
| Exon 13 6F | 5’AgTgATgCTgACTATgATTAC3’ | 56 | 3.5 | 478 |
| Exon 13 6R | 5’TgTgCTTTgCTgCTTgATC3’ |  |  |  |
| Exon 13 7F | 5’CAgATgTCAgggATACg3’ | 60 | 2.5 | 606 |
| Exon 13 7R | 5’ATAAAggAgCATggTgTgTg3’ |  |  |  |
| Exon 13 8F | 5’AAgTAAgACAggATggAggA3’ | 55 | 1.5 | 484 |
| Exon 13 8R | 5’AggAAggggACATTTgACT3’ |  |  |  |
| Exon 13 9F | 5’AgTCAAATgTCCCCTTCCT3’ | 60 | 2 | 766 |
| Exon 13 9R | 5’gACCAAAgTTTggggAAAgA3’ |  |  |  |
| Exon 13 0F | 5’TCTTTCCCCAAACTTTggTC3’ | 60 | 2 | 378 |
| Exon 13 0R | 5’ggCACATAATCAATTTCAgC3’ |  |  |  |

Abbreviations: (F)= forward (R)= reverse.
